# Preliminary study: Treatment with intramuscular interferon beta-1a results in increased levels of IL-12Rβ2^+ ^and decreased levels of IL23R^+ ^CD4^+ ^T - Lymphocytes in multiple sclerosis

**DOI:** 10.1186/1471-2377-11-155

**Published:** 2011-12-19

**Authors:** Jennifer M Kress-Bennett, Garth D Ehrlich, Ashley Bruno, J Christopher Post, Fen Z Hu, Thomas F Scott

**Affiliations:** 1 Center for Genomic Sciences, Allegheny-Singer Research Institute, 320 East North Avenue, Pittsburgh, PA 15212, USA; 2 Department of Microbiology and Immunology, Drexel University College of Medicine, Allegheny Campus, 320 East North Avenue, Pittsburgh, PA 15212, USA; 3 Department of Otolaryngology-Head and Neck Surgery, Drexel University College of Medicine, Allegheny Campus, 320 East North Avenue, Pittsburgh, PA 15212, USA; 4 Department of Neurology, Allegheny General Hospital, 320 East North Avenue, East Wing, Pittsburgh PA, 15212; USA; 5 Department of Neurology, Drexel University College of Medicine, Allegheny Campus, 320 East North Avenue, Pittsburgh, PA 15212, USA

## Abstract

**Background:**

There are a lack of biomarkers which can be used to predict clinical outcomes for multiple sclerosis (MS) patients receiving interferon beta (IFN-β). Thus the objective of this study was to characterize changes in CD4+ T-lymphocyte expression in an unbiased manner following initiation of intramuscular (IM) IFN-β-1a treatment, and then to verify those findings using marker-specific assays.

**Methods:**

Peripheral blood specimens were collected from twenty MS patients before and after treatment with intramuscular (IM) IFN-β-1a and were used for isolation of mononuclear cells (PBMCs). mRNA expression patterns of negatively-selected CD4+ T-cells from the PBMCs were analyzed using microarray gene expression technology. IL-12 and IL-23 receptor levels on PBMC-derived CD4+ T-cells were analyzed by flow cytometry. The phosphorylation status of Stat4 was measured by performing densitometry on western blots.

**Results:**

Microarray analyses demonstrated that mRNA expression of the IL-12Rβ2 gene was uniformly up-regulated in response to IFN-β-1a treatment and was associated with an increased number of IL-12Rβ2^+ ^CD4^+ ^T-cells by flow cytometry in 4 of 6 patients. This finding was substantiated by demonstrating that Stat4 phosphorylation, a transcription factor for IL-12, was increased after treatment. Conversely, the number of IL-23R^+ ^CD4^+ ^T-cells was decreased following treatment.

**Conclusions:**

The IL-12 receptor shares a common subunit, the IL-12Rβ2, with the IL-23 receptor. Both of these receptors have a probable role in regulating IL-17 and TH-17 cells, important mediators of inflammation in multiple sclerosis (MS). Thus, the changes in the numbers of CD4^+ ^T-cells expressing these receptors in response to IFN-β-1a treatment may point to an important mechanism of action for this drug, but further large scale studies are needed to confirm these preliminary observations.

## Background

Although interferon beta (IFN-β) therapies have been commonly used as a treatment for multiple sclerosis (MS) for over a decade, the mechanism of action (MOA) of these therapeutic agents remains largely undefined [1,2]. Given the central role of specific subsets of CD4^+ ^T-cells in the autoimmune process of inflammatory lesion formation in MS, a frequently proposed MOA for IFN-β's involves changes in activities of these cells. Although particular cytokines such as IL-12 and IL-23 and their receptors have been hypothesized to be likely mediators of inflammation in MS, details concerning receptor physiology in the treatment setting remain sparse [3,4]. Therapy induced changes in CD4^+ ^T-cell function have primarily been characterized in animal models of MS, and await verification in actual MS patients [5].

There is a pressing need for the identification of biomarkers that can be used early in a patient's treatment to estimate the efficacy of IFN-β therapies in the treatment of MS, as not all patients respond to this treatment modality. This need is becoming even more important as additional MS therapies become available, and as the goal of personalized medicine is to find a patient-specific therapy as early in treatment as possible to minimize additional relapses. Thus, the objective of this non-biased study approach, wherein we screened for changes in gene expression of thousands of genes, was to identify candidate biomarkers which can be tested in future large-scale population studies early in the treatment phase as a surrogate for efficacy.

## Methods

This research protocol was approved by the Allegheny-Singer Research Institute's Institutional Review Board (IRB). All study subjects were informed about the research protocol and voluntarily enrolled in the study by signing the IRB-approved consent forms.

### Samples

Three peripheral blood samples were obtained from each patient according to the following schedule: 1) pretreatment - the day prior to initiation of therapy with IFN-β-1a (Avonex^®^; 30 mg intramuscular); 2) 24 hours following initiation of therapy; and 3) 7 days following the initiation of therapy. These specimens were used for CD4+ T-lymphocyte gene expression studies for all twenty patients, and for a final group of six patients they were also used for fluorescence-activated cell sorting (FACS)-based cell surface marker studies

### Isolation of CD4^+ ^T-Cell mRNA

Mononuclear cells were purified from fresh peripheral blood specimens by centrifuging them onto a lymphocyte separation media (Ficoll-Paque: GE Healthcare; Piscataway, NJ) density step gradient. CD4^+ ^T-cells were purified from the mononuclear cell fraction by negative selection with magnetic beads (Miltenyi Biotec, Auburn, CA), and were subjected to lysis in Rneasy Lysis buffer (Qiagen; Valencia, CA). Messenger (m)RNAs were purified on oligo dT columns. Each mRNA sample was tested for purity and integrity by microcapillary electrophoresis using an Agilent Bioanalyzer (Santa Clara, CA). Any contaminating DNA detected by polymerase chain reaction (PCR)-based amplification of housekeeping genes, was removed by DNAse I treatment.

### Gene Expression analysis using microarrays

Microarray analyses were performed using Illumina's Expression Beadchip as previously described [6]. Biotinylated complementary (c) RNAs were prepared using 0.55 μg of total CD4+ T-cell mRNA using Illumina's TotalPrep RNA Amplification kit (San Diego, CA). Following fragmentation, 0.75 μg of each cRNA prep was hybridized to one of the bead chips. Arrays were scanned using the Illumina Bead Station and the resultant data were analyzed using Statistical Analysis of Microarrays (SAM) software, applied to individual (not pooled) specimens.

### Flow Cytometry

Specimens from the final six patients enrolled in the study were studied using flow cytometry. Their negatively-selected, CD4^+ ^T-cells were stimulated with phorbol 12-myristate 13-acetate (PMA, 10 ng⁄ml) and ionomycin (2 μmol/l) for 4 h in the presence of brefeldin A (10 ng ⁄ ml; Sigma). After stimulation the cells were washed with PBS and labeled with anti-CD4-FITC and anti-IL-23R-PE at 4°C for 30 min. The cells were then fixed and permeabilized prior to incubation with anti-IL-12R2-APC (R&D Systems, Minneapolis, MN) and immediately analyzed by flow cytometry. Data on 10,000 cell fluorescence events were acquired and analyzed using a FACSCalibur instrument (Becton-Dickinson, San Jose, Calif, USA), CellQuest™ and Paint-a-gate™ software.

### Immunoblotting

Total proteins were extracted from human CD4^+ ^T-cells negatively selected from the mononuclear white blood cell fraction as described above and quantified using a modified Bradford assay (Bio-Rad; Hercules, CA). Equal quantities of protein extract were resolved by 12% SDS-PAGE and transferred to nitrocellulose immobilization membranes. The membranes were probed with antibodies specific for IL-12Rβ2, IL-23R, Stat4 or phosphorylated Stat4, conjugated with HRP-labeled secondary antibody (R&D Systems, Minneapolis, MN), for signal detection followed by densitometry.

### Statistical Analysis

Data were expressed as the mean + SD. The difference between groups was analyzed by Student's *t *test, and *p *< 0.05 was considered statistically significant.

### Magnetic Resonance Imaging

MRI studies were performed prior to treatment and at one year post treatment. Cerebral axial T1- and T2-weighted imaging, and proton density and fluid attenuated inversion recovery (FLAIR) axial sagittal imaging was obtained for all patients. These were obtained on either a Siemens (Erlangen, Germany) 1.5 Magnetom units (D13B) or a General Electric (Milwaukee, WI) signa unit (1.5 Tesla) with 5-mm cuts and 5-mm gaps. All MRIs were reviewed by the senior investigator (T.S.).

## Results

We studied twenty relapsing-remitting MS patients (14 female and 6 male). All patients were sampled as describe in Methods. All patients were treatment naïve to IFN-β-1a (Avonex^®^; 30 mg intramuscular) and naive to all other MS therapies, with the exception of one patient who had remote prior exposure to glatiramir acetate. All patients were free of relapses for at least 60 days prior to enrollment in the study. The average age of the entire group was 37.1 years (range 26.6-57.5), with an average disease duration of 6.7 years (range 0.3-24.9). A subgroup consisting of the last 6 patients studied underwent more detailed study (see below) and consisted of 3 males and 3 females, average age 35.5 years (range 31.6-46.0), and average disease duration 4.5 years (range 0.5-25.5).

The results of the Illumina microarray experiments designed to examine gene expression changes in the CD4^+ ^T-cells of MS patients following initiation of IM IFN-β-1a (compared to pretreatment samples) were highly remarkable for a mean 30-fold increase in IL-12βR2 mRNA expression. We did not observe even 2-fold increases in gene expression for: IL-12, IL-12Rβ1, IL-23, or other IL-23 receptor proteins. In addition, no decreases in expression of 50% or greater following initiation of therapy were observed with the sole exception of the mRNA for IL23R gene.

**Figure 1:**
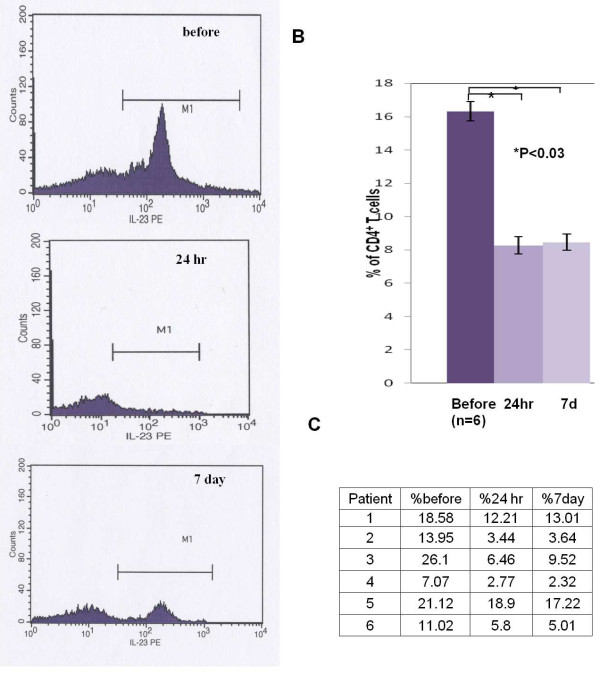
**Flow Cytometry of CD4^+ ^IL23R^+ ^T Cells. Expression of IL-23R subunit on the cell surface of CD4^+^T cells from MS patients treated with beta-interferon**. (A) Results of one representative patient. (B) Percentage of IL-23R^+ ^T cells from MS patients. Values are expressed as mean ± SEM. (C) Percentage of CD4^+^T cells expressing IL-23R in each MS patient treated with beta-interferon (n = 6).

**Figure 2:**
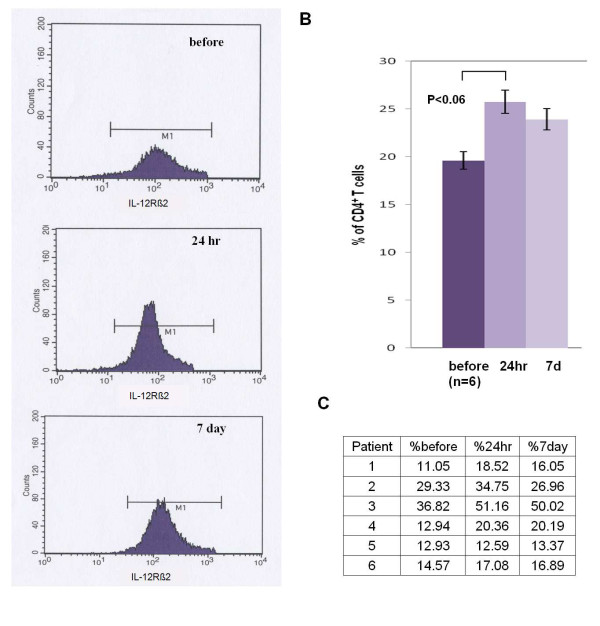
**Flow Cytometry of IL-12Rβ2^+ ^CD4^+ ^T cells from MS patients before, 24 hr and 7 d after treatment with beta-interferon**. (A) Results of one representative patient. (B) Percentage of IL-12Rβ2^+ ^T cells from MS patients shows that at 24 hours post treatment that there is an increase in this cell type that almost reaches statistical significance. Values are expressed as mean ± SEM. (C) Percentage of CD4^+^T cells expressing IL-12β2 in each MS patient treated with beta-interferon (n = 6).

Based on the results of the microarray data obtained on the first dozen patients enrolled in the study we designed flow cytometric assays to characterize the IL-23R and IL-12Rβ2 cell surface markers of CD4+ isolated from the PBMCs of the final six patients enrolled in the study. These analyses detected a significant decrease in IL-23R^+ ^CD4^+ ^T-cells in 6 out of 6 patients at both 24 hours and 7 days post initiation of IM IFN-β-1a treatment (Figure 1) (range 11-75% decrease versus pre treatment). In addition, 4 of the 6 patients examined by flow cytometry for their IL-12Rβ2^+^CD4^+ ^cells demonstrated increased numbers (range 16-65%) at both 24 hours and 7 days relative to the pretreatment levels, while one patient showed minimal changes (< 5% increase, patient 5) and the final patient had an increased ratio of IL-12Rβ2^+^CD4^+ ^cells at 24 hours, but not 7 days (patient 2)(Figure 2).

**Figure 3:**
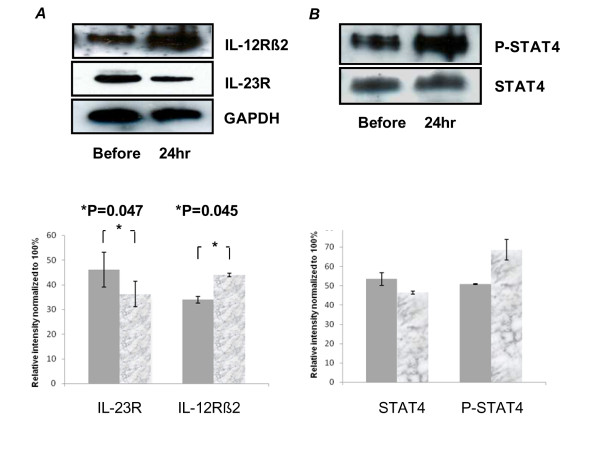
**IL-23R, IL-12Rβ2, STAT4 and phospho-STAT4 expression on CD4^+^T cells from MS patients treated with β-interferon**. Western blot analysis of (A) IL-23R and IL-12BR2 (B) pSTAT4 and STAT4 were performed by using total proteins extracted from CD4^+^T cells from three patients before and after treatment with B-interferon. In each panel a polyacrylamde gel shows the results of one representative experiment. Densiometry reports the relative intensity of all three patients. GAPDH was used as the internal loading control. (Data shown are all from the same patient).

Immunoblots were reliably produced for densitometry measurements of Stat4 and phosphorylated Stat4 on three of the six patients evaluated by FACS. Results for all three patients demonstrated an increase in phosporylated Stat4 after treatment (Figure 3). Blinded reviews of the charts of these 6 patients performed 12-16 months after initiation of IM IFN-β-1a therapy to determine disease activity revealed that none of the patients experienced relapses, received corticosteroids or developed any new MRI lesions.

## Discussion

This study was designed to broadly screen for early time-point changes in the gene expression profiles of CD4+ T-lymphocytes in MS patients receiving IFN-β therapy as a means to identify candidate biomarkers of response and nonresponse. The underlying hypothesis being that alterations in the immune functionality of CD4+ T-cells underlie the mechanism of action through which IFN-β-1a alters the development of the autoimmune lesions characteristic of MS. Our study was too small to provide a statistical correlate for lack of disease progression (typically defined by the absence both of relapsing events and the appearance of new MRI lesions during therapy), with specific biomarkers which could be identified early in the treatment regimen, however, the results of our experiments suggest that IM IFN-β-1a's efficacy in reducing the number of exacerbation events in MS may involve modulation of the number of IL-12 and IL-23 receptors on CD4^+ ^T-cells. This is particularly interesting as these two multi-protein receptors share a common subunit, the IL-12Rβ2. Thus, our findings suggest that IFN-β may alter the ratio of the other subunits of these two cytokine receptors although through what mechanism remains to be determined. Many other possible MOAs for IFN-β have been proposed, mostly focusing on inflammation (inhibition of leukocyte migration into the CNS, regulation of anti-inflammatory cytokines such as IL-4 and IL-10, and proposed pro-inflammatory cytokines such as IL-12, IL-17, and IL-23) [7-9]. One previous report, also starting with a nonbiased micro-array strategy, found an impressive induction of mRNA for IL-12Rβ2 in peripheral blood mononuclear cells of MS patients treated with IFN-β, and suggested a possible role of the IL-12 receptor (IL-12R) in the MOA of IFN-β [8]. A separate study reported that T-cells expressing the closely related receptor for IL23 were less prevalent in IFN-β treated patients [10]. Thus, our findings performed with purified CD4+ T-cells are in accord with both of these studies and support the concept that the T-cell response to IFN-β likely involves complex changes in the overall cytokine milieu and their receptors [4,11].

A recent study of IM IFN-β-1a-treated MS patients showed a decrease in serum IL-12 after chronic treatment, suggesting a possible dissociation between acute and chronic effects [4]. However, an *in vitro *study of stimulated CD45^+ ^T-cells showed no change in the percentage of cells expressing IL-12 receptors after treatment with IM IFN-β-1a [10], disagreeing with our *in vivo *results.

Recently, due to the development of multiple therapeutic agents for MS, there has been an emphasis placed on the development of biomarkers which could serve as early surrogates for the prediction of clinical response to treatment. These biomarkers would provide for rapid patient-specific tailoring of drug-treatment regimens to minimize the time a patient would remain on a therapy that would not be effective in ameliorating their disease. Such biomarkers should ideally be identifiable through a simple blood test. In terms of T-cell activities, there have been reports suggesting the use of increased serum levels of IL-17F [9], and MxA protein levels [12] as biomarkers for the effectiveness in IFN-β-treated MS patients. Our universal finding (6/6) of decreased numbers of IL-23R+ CD4+ T-cells, combined with an increased percentage of IL-12Rβ2^+ ^CD4^+ ^T-lymphocytes in most, but not all, IM IFN-β-1a-treated patients, none of which experienced measurable disease progression for a year or more following initiation of therapy, suggests that these cell surface markers should be further investigated in a large-scale prospective trial to determine their potential efficacy to serve as biomarkers of therapeutic effectiveness. It is important to stress that the number of patients in this study was too small to seriously consider attempting to identify a "responder" group. We did review the clinical status of our patients in terms of relapse and MRI changes, and found no difference in terms of patients with significant changes in IL-12Rβ2^+ ^CD4^+ ^cells versus those without.

We and others have previously used microarray mRNA expression experiments as an unbiased means to identify likely mediators of the therapeutic effect of IFN-β [6]. Although this technology has been criticized for being overly broad and merely exploratory, we find a combined approach, in which microarray surveys are used in the early stages of experimentation to provide candidate gene-protein systems for subsequent hypothesis-based characterization studies, serves as a powerful strategy to divine the mechanism of action of systemic therapeutics. This combined approach overcomes investigator bias that is associated with research that is purely hypothesis based and provides an attractive strategy for generating new discoveries with respect to the very large number of unknown cellular activities that still exist.

## Conclusions

In the current study we have observed significant shifts in the numbers of IL-12 and IL-23 receptors on CD4^+ ^T-cells in patients receiving IM IFN-β-1a therapy. Our finding of differential regulation of the IL-12 and IL-23 receptors among a small number of IM IFN-β-1a -treated patients warrants further investigation. The central role of IL-12 and IL-23 in the autoimmune process of MS suggests measurement of IL-12 and IL-23 receptor activities may lead to biomarkers for responsiveness to IFN-β.

## Competing interests

The authors declare that they have no competing interests.

## Authors' contributions

JB conceived study design and methodology, reviewed the paper and performed all assay work. GDE conceived study design and methodology, and reviewed the paper. AB conceived study design and methodology, reviewed the paper and performed assay work. JCP conceived study design and methodology, and reviewed the paper. FZH conceived study design and methodology, reviewed the paper, and supervised all assay work. TFS conceived study design and methodology, and reviewed the paper. All authors have read and approved the final version of this manuscript.

## Pre-publication history

The pre-publication history for this paper can be accessed here:

http://www.biomedcentral.com/1471-2377/11/155/prepub
